# Actionable Genomics in Clinical Practice: Paradigmatic Case Reports of Clinical and Therapeutic Strategies Based upon Genetic Testing

**DOI:** 10.3390/genes13020323

**Published:** 2022-02-10

**Authors:** Merlin G. Butler, Daniel Moreno-De-Luca, Antonio M. Persico

**Affiliations:** 1 Departments of Psychiatry & Behavioral Sciences and Pediatrics, University of Kansas Medical Center, Kansas City, KS 66160, USA; mbutler4@kumc.edu; 2 Genomic Psychiatry Consultation Service, Verrecchia Clinic for Children with Autism and Developmental Disabilities, Bradley Hospital, East Providence, RI 02915, USA; daniel_moreno_de_luca@brown.edu; 3 Division of Child and Adolescent Psychiatry, Department of Psychiatry and Human Behavior, Warren Alpert Medical School of Brown University, Providence, RI 02903, USA; 4 Department of Biomedical, Metabolic and Neural Sciences, University of Modena and Reggio Emilia, I-41125 Modena, Italy; 5 Child and Adolescent Neuropsychiatry Program, Modena University Hospital, I-41125 Modena, Italy

**Keywords:** 16p13.2 duplication, *COL3A1*, *DLG2*, Ehlers-Danlos syndrome, lithium

## Abstract

In clinical settings, the information provided by genetic testing can explain the triggers and processes underlying clinical presentations, such as neurodevelopmental disorders, in up to one third of affected individuals. However, translating this knowledge into better and more personalized clinical management to many appears a distant target. This article presents three paradigmatic cases to exemplify how this translational effort can, at least in some instances, be undertaken today with very positive results: (a) a young girl carrying a chr. 16p11.2 duplication can be screened using targeted exams and undertake therapeutic/preventive interventions related to her genetic diagnosis; (b) a 13-year-old boy with intellectual disability and autism spectrum disorder carries a chr. 11q14.1 deletion, partly spanning the *DLG2* gene important for synaptic function, and gained over 20 I.Q. points ostensibly due to carbolithium, prescribed in the absence of affective symptoms, exclusively following the pathophysiology pointed out by the genetic results; (c) a 58-year-old woman carries a *COL3A1* gene variant responsible for the vascular form of Ehler–Danlos syndrome with colon rupture. Detection of this variant in six members of her extended family allows for better clinical management of the proband and targeted genetic counselling for family members at risk of this connective tissue disorder. The unprecedented flow of genetic information available today through new technologies, if interpreted in the light of current knowledge in clinical diagnosis and care of those with connective tissue disorders and neurodevelopmental disturbances, in biology and in neuropsychopharmacology, can promote better clinical and pharmacological treatment, disease surveillance, and management provided and incorporated into the clinical setting.

## 1. Introduction

Many patients presenting for clinical evaluation due to complex conditions, including cardiovascular and neurodevelopmental disorders, are increasingly viewed as having a heterogeneous collection of genetic conditions, whose diagnosis is ultimately based on a predetermined set of shared features. In the case of autism spectrum disorder (ASD), for example, multiple lines of evidence support the convergence of these numerous, individually rare but collectively common genetic causes onto a relatively limited number of functional pathways, whose derangement affects neurodevelopment in ways which are beginning to be well understood at the molecular level through cellular and animal models [1]. However, the use of this genetic knowledge in the clinical management of children and adults with neurodevelopmental disorders is still not widely implemented. In the clinical routine, genetics is still often viewed as a super-specialized area of expertise somewhat collateral to the mainstream clinical management of patients with behavioral disorders. In an oversimplified view, genetic testing may be able to explain in up to one third of cases why a given child has received a clinical diagnosis of autism spectrum disorder (ASD), intellectual disability (ID), and so on, but the path for clinicians, patients, and families to translate these genomic results into better clinical management is often unclear. Without a strategy in place, patients will be prescribed the same behavioral intervention, speech or occupational therapy that would have been prescribed without any prior knowledge of genetic results. Similarly, genetic information will have little influence on psychopharmacological interventions, which remain non-disease specific, directly imported into child psychiatry from adult psychiatry, not always evidence-based and usually off-label [2,3]. In brief, many clinicians appear convinced that with or without positive genetic testing and consultation, the clinical management of children and adults with NDDs will essentially follow the same trajectory. Similarly, the application of advanced genetic testing (i.e., next-generation sequencing) in identifying affected or at-risk individuals for the presence of a potential life-threatening event, such as aortic aneurysm or internal organ rupture, is not part of the assessment typically performed in most clinical centers and yet, in the case with vascular Ehler–Danlos syndrome (EDS) which we shall describe, it can be instrumental in prioritizing life-saving strategies and management.

The number of studies addressing the genetic, molecular, and neurobiological underpinnings of behavioral and somatic conditions with high heritability as a whole and at the single-patient level has undergone exponential growth over the past twenty years. This, in turn, has produced a parallel growth of knowledge in the mechanisms underlying pathophysiology, spurring interest into the exploration of novel ways to translate this knowledge into better and more personalized clinical management, often labeled “precision medicine” [4]. This article presents three paradigmatic cases embodying this latter view, with the aim to show how it is already possible today, at least in some cases, to incorporate the information provided by diagnostic genetic testing into a personalized approach able to improve clinical practice in the areas of prognostic predictions and preventive follow-up, targeted medical testing and surveillance, and innovative and personalized psychopharmacology. These approaches will be a better support to families and patients. The three cases described in this article have been directly observed, diagnosed, and followed over time by the authors during medical care services. Informed consent was obtained from parents/patients for inclusion in studies at each of the respective diagnostic centers by the authors in accordance with their institutional review boards.

## 2. Actionable Genomics in Psychiatry: Example of a 16p11.2 Duplication Case Report

CNVs in 16p11.2 are among the most frequently identified pathogenic genetic changes in autism spectrum and other neurodevelopmental disorders [5,6]. They exemplify the variable expressivity and pleiotropy that tends to be the norm for high-risk genetic variants: deletions of this genomic interval are strongly associated with autism and intellectual disability [7], while duplications appear to increase risk more strongly towards psychotic disorders [8]. In addition to these neurobehavioral phenotypes, these genetic changes have an impact on other phenotypes outside the central nervous system that seem to be sensitive to gene dosage; for example, people with 16p11.2 deletions have a higher BMI and lower head circumference, while the opposite phenotypes are seen in those with 16p11.2 duplications [9]. Below, we report a clinical vignette that showcases how genetic information can be used in the clinic, highlighting decision points where management would differ based on genomic data. 

### Case Report 

We evaluated a now 16-year-old adopted female in our Genomic Psychiatry Consultation Service. She had a history of autism spectrum disorder, attention deficit and hyperactivity disorder, learning difficulties, trichotillomania, self-injurious behaviors, depressive disorder, and was presenting because of worsening anxiety. She had a family history of schizophrenia in her biological father, and mild intellectual disability in her biological mother. Genetic testing was obtained using a commercially available chromosomal microarray to detect deletions and duplications in the clinic setting due to her history of developmental delay, microcephaly, and ASD. It revealed a duplication of chromosome 16p11.2 (chr16:29560500-30104844 × 3, hg18). Regarding other medical comorbidities, she had agenesis of the corpus callosum, a mild distal low amplitude left hand intention tremor that she could suppress voluntarily, and no seizures or cardiac or renal anomalies were reported. A recent body mass index was 26.83, which lies at the 91st percentile for her age on the CDC growth curve for girls from 2–20 years. Her psychopharmacological regimen included sertraline for low mood and anxiety, aripiprazole for mood stabilization and irritability in the setting of an ASD, n-acetylcysteine for trichotillomania, and methylphenidate HCL for low attention. 

Recommendations in collaboration with her treating clinicians were made in the context of known behavioral and medical comorbidities associated with 16p11.2 duplications, many of which the patient was already displaying (microcephaly, ASD, ADHD, and learning difficulties). Regarding her medication regimen, we analyzed the risks and benefits particularly in the context of her rare genomic variant. She was already on aripiprazole, an antipsychotic medication that has an FDA indication for irritability in the setting of ASD, and which can also lead to weight gain, metabolic abnormalities, and decreased seizure threshold [3]. An EEG was obtained in the context of episodes of head tilting, repetitive picking, and decreased response to prompts, with her history of microcephaly and agenesis of the corpus callosum; it did not capture any clinical events, or evidence of seizure activity, diminishing our concern about this phenotype. Although she has a propensity for low BMI, she was already in the 91st percentile for BMI for her age, likely being protected from additional weight gain by her genomic profile, even when using one of the atypical antipsychotics with a more benign metabolic side effect profile. However, her genomic results also highlight that she is at increased risk for seizures, and aripiprazole can decrease the seizure threshold, making this an important consideration for monitoring in her case. Conversely, she also displayed ADHD, a phenotype frequently associated with 16p11.2 duplications [7]. ADHD has an excellent response to stimulant medications, which in turn can also decrease appetite and lead to weight loss; had it not been for her current higher BMI, we would have had to pay particularly close attention given her propensity to leaner BMI because of her 16p11.2 duplication, and even consider other second line strategies if this were observed, such as alpha agonists. In addition, although structural cardiac abnormalities are seen less frequently in people with 16p11.2 duplications, they can be present, and screening for these before starting medications like stimulants, which can lead to increased risk for cardiac arrhythmias in people with congenital heart anomalies. In addition, given her high risk for schizophrenia, it is particularly important to continue active surveillance for prodromal psychotic symptoms, especially as she enters young adulthood. Based on evidence of other rare genetic conditions at ultra-high risk for psychosis, we also recommended starting omega-3 fatty acids, given their favorable risk:benefit profile [10], and their additional effect on dampening potential hypertriglyceridemia stemming from atypical antipsychotic use, like the patient’s case. 

From a behavioral standpoint, it was helpful to take into context the language impairments that have been previously associated with 16p11.2 duplications to ensure that academic strategies were adequately identifying her areas of relative non-verbal cognitive strengths, to target the delivery of interventions by using alternative means of communicating these. Given her susceptibility to psychosis and the beneficial effect of cognitive-behavioral therapy (CBT) in preventing transition to psychosis in high-risk individuals [11], we recommended using this treatment as the modality of choice. 

Lastly, from a social support standpoint, we were able to provide parents with additional phenotypic and natural history information about 16p11.2 duplications from multiple resources written in lay language and with contact information for support groups of other families and individuals with 16p11.2 duplications (https://www.rarechromo.org/media/information/Chromosome%2016/16p11.2%20Microduplications%20QFN.pdf, accessed on 7 February 2022). Access to these resources may decrease the feelings of isolation that families with rare genetic disorders often have. We recommended following up with genetic counseling within our team in the future to discuss family planning implications, when and if this becomes relevant for her. Her longitudinal care in our clinic has evidenced an overall decrease in her anxiety and behavioral outbursts, with occasional episodes of anxiety in response to environmental stressors that we addressed with treatment followed by a return to her baseline mood. She has also maintained a stable weight, and has been meeting her academic goals successfully in the framework of her established individualized education plan.

## 3. Genetically-Driven Psychopharmacotherapy: A Paradigmatic Case of Intellectual Disability and Autism Spectrum Disorder

The psychopharmacotherapy of ASD and intellectual disability represents a challenging area for clinicians working in the field of neurodevelopmental disorders. On the one hand, pharmacological treatments currently available to treat ASD and ID are not directly effective on core symptoms, but rather on comorbid symptoms and problematic behaviors, such as sleep disorders, hyperactivity, anxiety, depression, irritability, aggression, self-injurious behavior, and epilepsy [2,3,12]. On the other hand, patients with neurodevelopmental disorders display much greater interindividual variability in response to psychoactive drugs and side effect sensitivity, as compared to patients with other psychiatric and behavioral disorders [3]. Genetic testing, paired with in-depth knowledge of developmental neuroscience and psychopharmacology can begin to provide novel and personalized treatment algorithms. We hereby report a case whose drug treatment was chosen based on the results of genetic testing, yielding an unprecedented success in terms of cognitive improvement. 

### Case Report 

A.G.Z. is a boy first observed at 13 years of age, when he was diagnosed with «Mild intellectual disability, autism spectrum disorder (severity level 1) and language disorder» according to DSM-5 diagnostic criteria [13]. A mild global developmental delay was observed in early childhood, especially for motor milestones (walking at 18 months) and for expressive language development (single words at 24 months, simple sentences at 4 years). He had difficulties interacting with peers both in school and in play contexts. Hand motor stereotypies and repetitive jumping were observed when the child was excited. At 11-years-old, cognitive testing using the WISC-III yielded a Total I.Q. of 55, Verbal I.Q. 59 and Performance I.Q. 62. Starting at age 12 years, parents noticed a slowly progressive cognitive worsening, especially in reference to memory, with increasing difficulty acquiring new information and lack of consolidation of recently acquired practical skills. This patient was followed up for a total of 6 years, first at the Child and Adolescent Neuropsychiatry Unit at University Campus Bio-Medico (Rome, Italy) and subsequently at the Interdepartmental Program “Autism 0–90” of the “G. Martino” University Hospital in Messina (Italy).

**Figure 1:**
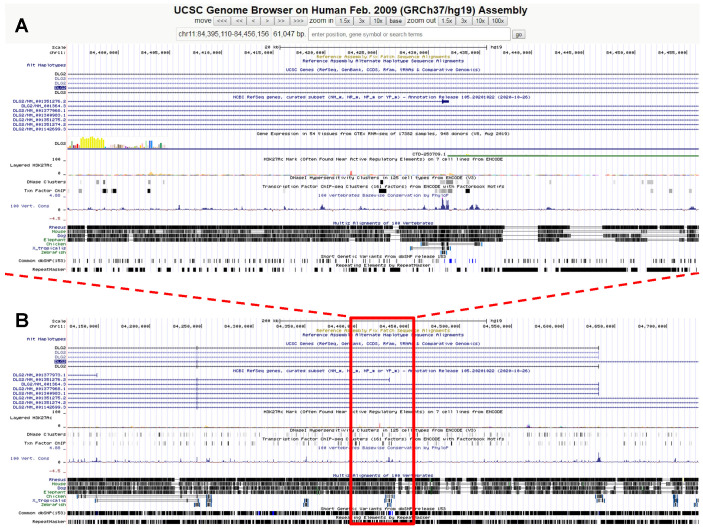
UCSD Genome browser views of (**A**) the 61 kb deletion located in chr. 11q14.1 carried by A.G.Z. (chr11:84,395,110–84,456,156/hg19), and (**B**) a 10X enlargement showing the surrounding genomic context. The deletion falls within the *DLG2* gene (“discs large MAGUK scaffold protein 2”), spanning the highly conserved exon 1 of transcript variant 9.

Genetic testing by array-CGH, using the Human Genome CGH SurePrint G3 Microarray 4 × 180 K Kit (Agilent Technologies, Santa Clara, CA, USA), unveiled a rare, maternally inherited deletion located in chr. 11q14.1 (Figure 1). This small deletion, spanning 61 kb, involves the highly conserved exon 1 of transcript variant 9 of the *DLG2* gene (“discs large MAGUK scaffold protein 2”) (Figure 1). *DLG2* is primarily expressed in the central nervous system (CNS), especially in the neocortex, hippocampus, and basal ganglia, and at the subcellular level in the somatodendritic compartment of neuronal cells [14]. Multiple transcript variants using alternative transcription start sites generate different DLG2 protein isoforms expressed in different neuronal cell types. DLG2, also known as PSD-93 or Chapsyn-110, belongs to the membrane-associated guanylate kinase (MAGUK) family, including also DLG4, also known as PSD-95 [15]. DLG2, dimerizing with DLG4, anchors NMDA glutamate receptors, K^+^ channels, and various intracellular signaling proteins to the scaffold of the post-synaptic glutamatergic element, keeping receptors clustered together within specific synaptic hotspots [14,15,16]. Derangement of this mechanism is detrimental to hippocampal long-term potentiation [17]. DLG2 also interacts with neuroligins [15] and megalin [18]. *DLG2* mutant mice display deficits in some forms of associative learning [19], and autism-like behaviors, including decreased sociability and increased repetitive self-grooming [20]. Rare disruptive *DLG2* gene variants are associated with a variety of neurodevelopmental disorders, including schizophrenia, intellectual disability ADHD, and autism spectrum disorder [21,22,23,24,25,26,27,28,29], as well as with bipolar disorder [30]. The SFARI gene database (https://gene.sfari.org, accessed on 7 February 2022) lists *DLG2* and its binding partner *DLG4* as «autism genes» with scores of 2 and 1 (i.e., “strong candidate” and “high confidence”), respectively.

Several points summarized above, namely (a) the strong functional link between DLG2 and NMDA glutamate receptors [14], (b) the role of DLG2 in associative learning [17,19], (c) the association of *DLG2* with cognitive and neurodevelopmental disorders [21,22,23,24,25,26,27,28,29], and (d) the decreasing memory performance observed in our patient at early adolescence, when the brain physiologically undergoes a massive pruning of glutamatergic synapses [31], all led to the suggestion that therapies able to boost NMDA receptor function might have been beneficial to our patient. The first strategy we considered was to increase AMPA glutamate receptor activity, in order to remove more frequently the blockade of NMDA receptors exerted by Mg^++^ ions, thus promoting cation influx through the NMDA channel pore [32]. Initially ampakines were considered, but CX717 was not made available for compassionate use in our patient by the producing company. Turning to psychoactive drugs more broadly available in the European market, lithium appeared as the best candidate at the time. In fact, several studies were already indicating that by elevating δ-catenin, lithium could influence the clustering of AMPA receptors in the post-synaptic element of glutamatergic synapses, actively modulating neuronal activity [33]. More recently, lithium has been convincingly shown to enhance regular syncronous neuronal activity at therapeutic doses, whereas it produces epileptiform burst-like activity at overdose concentrations: both these effects are AMPA receptor-mediated and indeed lithium-induced epileptiform discharges can be effectively blocked by the AMPA receptor antagonist perampanel [34]. Collectively these studies point toward lithium as a potentially beneficial drug for this patient. Carbolithium was thus prescribed and titrated up to 450 mg b.i.d., yielding serum levels of approximately 0.50 mEq/L which were stably maintained throughout the duration of treatment, as verified every 3–6 months. These serum levels, which represent the lower limit for affective disorders, were chosen in agreement with the family, both to minimize the probability of side-effects and because the therapeutic range for affective disorders may not have applied to this novel indication. 

**Figure 2:**
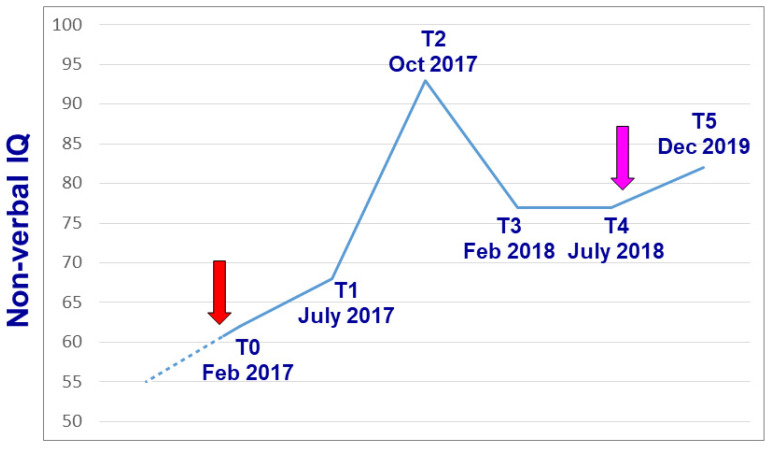
Variation in global non-verbal I.Q. over time, as recorded using the Leiter-R scale [35]. The beginning and the end of lithium treatment are indicated by the red and purple arrows, respectively. The hyphenated line connects T0 with a prior I.Q. measurement performed elsewhere at age 11 using the WISC-III scale (see text).

Lithium was started when the patient was 15 years of age. Cognitive functions were then periodically assessed using the Leiter-R [35], in order not to penalize the patient due to his expressive language impairment. His non-verbal I.Q. improved over the following months, reaching the maximum score of 94 after 8 months of low-dose lithium maintenance treatment (Table 1, Figure 2). In addition, selective visual attention steadily improved, as assessed using the Bell test [36] (Table 1). During the course of this cognitive improvement, both parents observed that the boy achieved greater adaptive functions and, sadly, greater insight into his condition. In fact, after one year of lithium therapy he suddenly asked his mother «Why am I different from all my classmates? ». Up until then, his parents claimed that he never realized the consequences of his intellectual disability, social difficulties, and limited communication skills. The achievement of this greater insight was followed by approximately one month of depressed mood, with subsequent recovery of normal mood and adaptive level. The last psychodiagnostic assessment performed under lithium treatment yielded a steady non-verbal I.Q. of 77 (July 2018 in Table 1). In September 2018, lithium was spontaneously stopped by the patient in agreement with his family, without notifying the prescribing physician. I.Q. testing performed more than one year after the interruption of lithium treatment yielded a non-verbal I.Q. of 84 (Table 1). 

## 4. COL3A1 Gene Variant and Rupture of the Colon in an Extended Family with a Connective Tissue Disorder

Ehlers–Danlos syndrome (EDS) is included in a group of heterogeneous connective tissue disorders caused by single gene defects classified into 13 subtypes with 19 recognized dominant or recessive genes involved in their clinical presentations as listed in Table 2. Based on the 2017 international classification of Ehlers–Danlos syndrome, the 13 subtypes include classical, classical-like, cardiac-valvular, vascular, hypermobile, arthrochalasia, dermatosparaxic, kyphoscoliotic, brittle cornea syndrome, spondylodysplastic, musculocontractural, myopathic, and periodontal [37,38]. Most of the types of EDS have hypermobility in common, including joint dislocations/subluxations or other musculoskeletal concerns with stretchable skin, blood pressure instability, or cardiovascular problems including pain [39]. Classical EDS is characterized by joint hypermobility, easy bruising and loose skin with poor wound healing and atrophy and occurs between 1 in 5000 to 1 in 20,000 individuals [37]. The genes disturbed in classical EDS are COL5A1, COL5A2, and COL1A1. Features of this syndrome may overlap with the vascular form of EDS, including a high risk of arterial or aortic aneurysms, gastrointestinal perforation, and uterine rupture during pregnancy due to COL3A1 gene defects [40]. The relationship and delineation of EDS subtypes requires detailed genetic testing with next-generation sequencing (NGS), use of bioinformatics, and searching genomic databases. In addition to exome and genome sequencing, also disease-specific gene panels with over 75 genes for connective tissue disorders are now available for testing in the clinical setting, using services from commercially available laboratories. Genetic results in this context can impact diagnosis, prognosis, disease surveillance and monitoring, as well as genetic counseling with evaluation of at-risk family members, having a 50% risk for autosomal dominant gene defects in first degree relatives. Individuals with vascular EDS (vEDS) are at risk of having arterial and/or aortic aneurysms in addition to internal organ rupture [37,38], as seen in several members within this family. Vascular EDS is an autosomal dominant disorder and a 50% risk for each of her first-degree relatives. Our proband and her at-risk family members including first degree relatives (siblings, children and parents) should be monitored or screened using ultrasound or CT scans and echocardiograms to determine the presence or absence of life-threatening events (e.g., aortic aneurysm) at baseline regardless of clinical symptoms, particularly if they are found to carry the same genetic defect. These patients should be monitored closely depending on the image results. Using regular surveillance scans to determine its size, location, and instability for surgical procedures would be needed to treat or avoid mortality if an aortic aneurysm is detected. In addition, medical treatment may be undertaken that allows restriction of blood pressure and heart function to avoid increased risk of an aneurysm if indicated to further increase viability beyond the direct colonostomy concerns raised to monitor for early signs of colon damage prior perforation, as in our probands and family members. Uterine rupture risks should also be discussed with reproductive age females as consequences of vEDS and pregnancies monitored closely. In this report we describe a proband with a variant of the COL3A1 gene causing vascular EDS with six males and females affected with spontaneous perforation of the colon over three generations in an extended family.

### Case Report

C.P. is a 58-year-old female seen in the Genetics Clinic at the University of Kansas Medical Center for discomfort in the upper quadrant of her abdomen, irritable bowel syndrome (IBS), and a history of surgical repair during the past two years due to a spontaneous transverse colon perforation without diverticulosis and a positive family history of colon perforation. She had no history of Hirschsprung’s disease or megacolon prior to colon perforation. She had a previous history of abdominal pain and discomfort as a young child and IBS, fibromyalgia, and Raynaud’s phenomenon since 18 years of age. Other review of systems was normal. The family history showed her maternal grandmother dying at 40 years of age from an unknown cause possibly involving the female reproductive tract. Her mother had a perforated colon at age 78 years and the surgical repair was attempted with some success but died two years later from poor healing and leakage from the intestine. Her 53-year-old sister had a colon resection due to imminent perforation and is under surveillance. Her 61-year-old sister had a perforation of the colon with colostomy and is under surveillance. Her maternal niece had a colon perforation age 31 years of age and is under current surveillance. Her first cousin (her mother’s brother’s son) was diagnosed with a perforation of the colon at six years of age and has a colostomy. Hence, there are six affected individuals including both sexes having had a perforation of the colon and/or surgical procedures performed due to an imminent risk of colon perforation.

Due to the extended family history of colon perforation in our proband and other family members, a connective tissue disorder gene panel consisting of 74 genes was ordered through a CLIA approved commercial laboratory using next-generation sequencing (NGS) with DNA isolated from saliva and buccal cells from our proband. The DNA results showed a variant of the autosomal dominant *COL3A1* gene with 51 exons located on chromosome 2 at DNA coding sequence c.1961G>A; p.Gly654Glu and classified as likely pathogenic. This variant is located in the collagen triple helix repeat domain (residues 168–1196) and disrupts the Gly-X-Y motif. Numerous similar amino acid changes within this highly conserved protein motif have been reported in individuals with vascular Ehlers–Danlos syndrome [41,42]. This variant has not been reported in the Broad EXAC dataset. It has an amino acid conservation indicating that the wild-type amino acid is conserved in all 62 mammalian DNA examined with a G at position 1961; all ten computer predictive algorithms generated a deleterious change, and the Grantham distance was 98 indicating a moderate change. Hence, the laboratory-based evidence supported a likely pathogenic variant. Two of her adult daughters showed the same COL3A1 gene variant and features of a connective tissue disorder including hyperflexibility and joint pain. Other at-risk family members are scheduled to be tested and a surveillance follow-up has been planned, including non-invasive gastrointestinal imaging for colon diverticuli or other anatomical findings indicating areas of weakness, and function status.

For a *COL3A1* pathogenic variant, in general major complications are seen in 15% of patients prior to genetic testing with a median survival of 50 years [43,44,45]. About 60% of patients are diagnosed prior to age 18 years. There are over 600 unique COL3A1 gene variants reported while the null variants, which are missing a protein product, have a milder presentation, and lower risk of bowel perforation or other major complications with increased life expectancy. The penetrance appears to be close to 100% in adults with missense or exon-skipping alterations. Gastrointestinal perforations generally involve the sigmoid colon and present in about 15% of individuals with a pathogenic variant but seldom in those with null gene variants which holds true in our family with a missense change [45]. Small bowel or stomach ruptures are less common. Bowel rupture is rarely lethal (3%) [42] with most deaths from complications of surgery, e.g., hemorrhage or poor tissue repair or healing. Iatrogenic perforation during colonoscopy has been reported [46] and surveillance should be carefully planned and followed.

## 5. Discussion

The three cases reported in this article show how key genetic information obtained through array-based and NGS technologies, paired with a clinical consultation within the framework of a multidisciplinary approach to medical and neurodevelopmental conditions, can have a direct impact on patient management by: (a) increasing awareness about potential medical comorbidities that may be currently present and yet to be uncovered, or for which patients may be at increased risk in the future; (b) providing additional information about the natural history based on other patients with the same rare genetic changes, especially as it relates to diagnostic conceptualization of neuropsychiatric phenotypes; (c) providing additional resources and connecting families with support organizations specifically focused on their rare genetic change, diminishing the isolation that is often felt by families with rare genetic conditions; (d) offering additional information for family planning; (e) putting an end to a diagnostic odyssey and avoiding unnecessary invasive testing, while simultaneously focusing healthcare resources and imaging on uncovering potential comorbidities; (f) making informed or even targeted neuropsychopharmacological interventions, based on underlying neurobiological mechanisms and/or known risk profiles. Even the prescription of psychotherapy may benefit in some cases from genetic information, in reference both to indication (par. 2 here) and possibly to treatment of choice [47]. 

The cases carrying a 16p11.2 duplication and an inherited missense variant in the *COL3A1* gene, described in par. 2 and 4, respectively, highlight the clear benefits of genetic information in the medical evaluation process and prognostic management of these patients. Detecting a duplication in chr. 16p11.2 allowed targeted medical testing to monitor propensity for seizures and to promote better pharmacological management in reference to psychostimulant prescription. Preventive administration of omega-3 fatty acids and CBT was undertaken to counteract the vulnerability to develop psychotic ideation, associated with 16p11.2 duplication. For the *COL3A1* pathogenic variant, invasive colonoscopy was avoided both for the patient and for other family members carrying the same variant, as it can lead to iatrogenic colon damage and/or perforation in these patients [46]. Importantly, colonoscopy would have likely been performed as a routine diagnostic procedure given the patient’s age and symptoms, were it not for the genetic results. Non-invasive surveillance for the patient, her two daughters, and other family members was also planned accordingly. Moreover, in the case illustrated in par. 3, lithium represents an example of a personalized therapy prescribed exclusively on the basis of the pathophysiological framework derived from array-CGH analysis. In fact, patient and family history were absolutely negative for bipolar disorder, major depression, or other psychiatric disorders for which lithium is typically prescribed. All psychodiagnostic measurements performed since childhood prior to lithium treatment, yielded an I.Q. or D.Q. <65. During one year and a half of lithium treatment, a consistent and long-lasting improvement in cognitive functions, visual attention, adaptive functioning, and motivational drive was both recorded using psychodiagnostic testing (Table 1, Figure 2) and observed clinically. Noticeably, this improvement was still detectable more than one year after lithium was stopped (Figure 2). This outcome appears unlikely to represent a chance finding and is even more encouraging, if we consider that at the beginning of adolescence, parents were observing a decrease in memory and cognitive performance. Two separate anedoctal observations describe lithium treatment as reverting cognitive regression in patients with Phelan–McDermid syndrome due to haploinsufficiency of the *SHANK3* gene [48,49]. Effective lithium blood levels were slightly higher in these cases than the ones proven effective in our patient (0.7–0.8 vs. 0.5 mEq/L). Each observation of a single case remains anedoctal and speculative until replicated in a patient sample, because an unusually benign spontaneous evolution not related to lithium treatment, though unlikely, cannot be excluded. However, our case report and the two cases with Phelan–McDermid syndrome described above [48,49], collectively spur interest into exploring the possible therapeutic efficacy of lithium in larger samples of patients with autism and/or intellectual disability due to disruptive genetic variants affecting *DLG2* [29] or other genes encoding postsynaptic glutamatergic scaffolding proteins [50].

## 6. Conclusions

We hope that case descriptions such as the ones reported in this article, which emphasize the complexities and “real world” presentation of people with psychiatric or unusual medical complications and conditions, can showcase how actionable clinical interventions based on genomic data can be put in place now, with readily available resources. Although exciting new discoveries offer the potential of directly targeting the molecular underpinnings altered in rare genetic disorders, incorporating genomic data for clinical treatment in the same way that medicine factors in multiple other sources of clinical data, can lead to less missed opportunities to improve our patients’ health, which is the ultimate goal of precision medicine.

## Figures and Tables

**Table 1:** Results of repeated psychodiagnostic measurements of non-verbal-I.Q. (Leiter-R) [35] and selective visual attention (Bell test) [36]. Lithium was started immediately after the February 2017 testing and was spontaneously stopped by the patient in September 2018.

		February 2017	July 2017	October 2017	February 2018	July 2018	December 2019
Leiter-R	Global I.Q.	61	69	94	77	77	82
	Fluid Intelligence	77	69	84	71	80	73
	Brief I.Q.	60	68	83	71	82	74
Bell test	Accuracy	−4.5 s.d.	−2.5 s.d.	−0.62 s.d.	-	-	-
	Speed	−1.8 s.d.	−1.13 s.d.	+0.09 s.d.	-	-	-

**Table 2:** Classification and genetic information about Ehlers–Danlos Syndrome (EDS) (from ref. [37] modified).

EDS Subtype	Gene (Protein)	Inheritance
Classical EDS (cEDS)	Major: *COL5A1* (COLLAGEN, TYPE V, α-1) Major: *COL5A2* (COLLAGEN, TYPE V, α-2) Rare: *COL1A1* (COLLAGEN, TYPE I, α-1)	AD
Classical-like EDS (clEDS)	*TNXB* (TENASCIN XB)	AR/AD?
Vascular EDS (vEDS)	Major: *COL3A1* (COLLAGEN, TYPE III, α-1) Rare: *COL1A1* (COLLAGEN, TYPE I, α-1)	AD
Kyphoscoliotic (kEDS)	*PLOD1* (PROCOLLAGEN-LYSINE, 2-OXOGLUTARATE 5-DIOXYGENASE) *FKBP14* (FK506-BINDING PROTEIN 14)	AR
Brittle cornea syndrome (BCS)	*ZNF469* (ZINC FINGER PROTEIN 469) *PRDM5* (PR DOMAIN-CONTAINING PROTEIN 5)	AR
Periodontal EDS (pEDS)	*C1R* (COMPLEMENT COMPONENT 1, r SUBCOMPONENT) *C1S* (COMPLEMENT COMPONENT 1, s SUBCOMPONENT)	AD
Anthrochalasia EDS (aEDS)	*COL1A1* (COLLAGEN, TYPE I, α-1) *COL1A2* (*COLLAGEN, TYPE I, α-2)*	AD
Musculocontractural EDS (mcEDS)	*CHST14* (CARBOHYDRATE SULFOTRANSFERASE 14) *DSE* (DERMATAN SULFATE EPIMERASE)	AR
Myopathic EDS (mEDS)	*COL12A1* (COLLAGEN, TYPE XII, α-1)	AD/AR
Cardiac-valvular EDS (cvEDS)	*COL1A2* (COLLAGEN, TYPE I, α-2)	AR
Spondylodysplastic EDS (spEDS)	*B4GALT7* (β-1,4-GALACTOSYLTRANSFERASE 7) *B3GALT6* (β-1,3-GALACTOSYLTRANSFERASE 6) *SLC39A13* (SOLUTE CARRIER FAMILY 39-ZINC TRANSPORTER, MEMBER 13)	AR
Dermatosparaxis EDS (dEDS)	*ADAMTS2* (A DISINTEGRIN-LIKE AND METALLOPROTEINASE WITH THROMBOSPONDIN TYPE 1 MOTIF, 2)	AR
Hypermobile EDS (hEDS)	Unknown	AD?

AR = autosomal recessive, AD = autosomal dominant.

## Data Availability

No data were generated.
